# Nitric oxide activates ATP-sensitive potassium channels in mammalian sensory neurons: action by direct S-nitrosylation

**DOI:** 10.1186/1744-8069-5-12

**Published:** 2009-03-14

**Authors:** Takashi Kawano, Vasiliki Zoga, Masakazu Kimura, Mei-Ying Liang, Hsiang-En Wu, Geza Gemes, J Bruce McCallum, Wai-Meng Kwok, Quinn H Hogan, Constantine D Sarantopoulos

**Affiliations:** 1 Department of Anesthesiology, Medical College of Wisconsin, 8701 Watertown Plank Road, Milwaukee, WI 53226, USA; 2 Department of Anesthesiology, Sakaide Kaisei Hospital, Sakaide, Kagawa, Japan; 3 Department of Pharmacology & Toxicology, Medical College of Wisconsin, 8701 Watertown Plank Road, Milwaukee, WI 53226, USA

## Abstract

**Background:**

ATP-sensitive potassium (K_ATP_) channels in neurons regulate excitability, neurotransmitter release and mediate protection from cell-death. Furthermore, activation of K_ATP _channels is suppressed in DRG neurons after painful-like nerve injury. NO-dependent mechanisms modulate both K_ATP _channels and participate in the pathophysiology and pharmacology of neuropathic pain. Therefore, we investigated NO modulation of K_ATP _channels in control and axotomized DRG neurons.

**Results:**

Cell-attached and cell-free recordings of K_ATP _currents in large DRG neurons from control rats (sham surgery, SS) revealed activation of K_ATP _channels by NO exogenously released by the NO donor SNAP, through decreased sensitivity to [ATP]i.

This NO-induced K_ATP _channel activation was not altered in ganglia from animals that demonstrated sustained hyperalgesia-type response to nociceptive stimulation following spinal nerve ligation. However, baseline opening of K_ATP _channels and their activation induced by metabolic inhibition was suppressed by axotomy. Failure to block the NO-mediated amplification of K_ATP _currents with specific inhibitors of sGC and PKG indicated that the classical sGC/cGMP/PKG signaling pathway was not involved in the activation by SNAP. NO-induced activation of K_ATP _channels remained intact in cell-free patches, was reversed by DTT, a thiol-reducing agent, and prevented by NEM, a thiol-alkylating agent. Other findings indicated that the mechanisms by which NO activates K_ATP _channels involve direct S-nitrosylation of cysteine residues in the SUR1 subunit. Specifically, current through recombinant wild-type SUR1/Kir6.2 channels expressed in COS7 cells was activated by NO, but channels formed only from truncated isoform Kir6.2 subunits without SUR1 subunits were insensitive to NO. Further, mutagenesis of SUR1 indicated that NO-induced K_ATP _channel activation involves interaction of NO with residues in the NBD1 of the SUR1 subunit.

**Conclusion:**

NO activates K_ATP _channels in large DRG neurons via direct S-nitrosylation of cysteine residues in the SUR1 subunit. The capacity of NO to activate K_ATP _channels via this mechanism remains intact even after spinal nerve ligation, thus providing opportunities for selective pharmacological enhancement of K_ATP _current even after decrease of this current by painful-like nerve injury.

## Background

Nitric oxide (NO) is a pivotal signaling molecule involved in many diverse developmental and physiological processes in the mammalian nervous system [1,2]. The influences of NO upon nociceptive transmission are opposing and complex [3-8], and the exact sites and mechanisms of these actions remain controversial. For example, within the spinal cord, high concentrations of NO exaggerate pain sensitivity [6], and pharmacological inhibition or genetic deletion of nNOS diminish pain behavior in several animal pain models [3,4,6,8,9]. Furthermore, expression of nNOS in sensory neurons is up-regulated following peripheral nerve injury [3,5,10], suggesting a contribution of NO to neuropathic pain. There is also evidence that NO has analgesic effects. Specifically, NO donors produce peripheral antinociceptive effects in inflammatory pain [11]. Also, low concentrations of NO acting at spinal sites attenuate allodynia following nerve injury [7,11,12]. These divergent findings reflect the site-specific complexity of NO-dependent signaling in the regulation of pain generating processes. Additionally, the NO-signaling pathway contributes to the anti-nociceptive effect of drug action at peripheral transduction sites, including that of opioids, NSAIDs, and the NO-releasing derivative of gabapentin NCX 8001 [13-16]. Some drugs produce peripheral analgesia via NO-dependent activation of ATP-sensitive potassium (K_ATP_) channels [15,17-19].

K_ATP _channels, widely represented in metabolically active tissues, are hetero-octamers composed of four regulatory SUR subunits (SUR1, SUR2A, or SUR2B) and four ATP-sensitive pore-forming inwardly rectifying potassium channel (Kir6.x) subunits (Kir6.1 or Kir6.2) [20]. Because their opening is determined by the cytosolic ADP/ATP ratio, K_ATP _channels act as metabolic sensors, linking cytosolic energetics with cellular functions in various tissues [21,22]. In the central and peripheral nervous system, widely distributed K_ATP _channels [20,23-25] regulate neuronal excitability, neurotransmitter release, ligand effects, and cell survival during metabolic stress [21,22,24,26,27].

NO regulates K_ATP _channels that control various physiological functions, including NO-associated protection from cell death, vasodilatation, and modulation of transmitter secretion [21,22,24,26]. Therefore, we hypothesized that NO activates K_ATP _currents in peripheral sensory neurons.

Altered sensory function contributes to the pathogenesis of neuropathic pain via hyperexcitability in injured axons [28-30] and the corresponding somata in the DRG [29,31], increased synaptic transmission at the dorsal horns [32], and loss of DRG neurons [33,34]. We have recently identified loss of K_ATP _currents in large DRG somata from rats that demonstrated sustained hyperalgesia-type response to nociceptive stimulation after axotomy [25,35]. Thus, reduced K_ATP _currents may be a factor in generating neuropathic pain through increased excitability, amplified excitatory neurotransmission, and enhanced susceptibility to neuronal cell death. Therefore, we also hypothesized that altered NO regulation may account for the decreased K_ATP _channel opening following axotomy that mediates the injury effect.

Since somata of injured DRG neurons are a site of pertinent phenotypic changes, reduced K_ATP _currents may contribute to neuropathic pain by increasing excitability, excitatory neurotransmission, and susceptibility to neuronal cell death. The established regulation of excitability by K_ATP _currents raises the hypothesis that decreased NO regulation may account for the decreased K_ATP _channel opening following axotomy that leads to neuropathic pain. Ionic channel modulation by NO can be produced either indirectly through the classical pathway of sGC activation and generation of cGMP, or directly through a pathway involving S-nitrosylation of target proteins [1]. S-nitrosylation regulates Na^+ ^channels and acid-sensing ion channels in DRG neurons [36,37], but effects on DRG neuronal K_ATP _channels have not been examined. It is also controversial whether the classical NO/sGC/cGMP/PKG pathway functions in DRG neurons. Specifically, pharmacological studies *in vivo *imply that K_ATP _channels in peripheral sensory neurons may be activated indirectly via the NO/cGMP/PKG pathway [38-40]. Also, the PDE inhibitor sildenafil increases cytosolic cGMP producing peripheral analgesia via activation of the NO/cGMP/PKG pathway [41]. Molecular constituents of this classical indirect pathway have been identified in mammalian DRG neurons, including NOS [3,5,10], sGC [42,43], cGMP [44] and PKG [45]. However, other published reports failed to find cGMP activity in DRG neurons, even after the up-regulation of NOS following axotomy or perfusion with NO donors [46]. Also a recent study failed to demonstrate sGC in mouse DRG neurons [45]. Because of these various controversies, we designed additional experiments to identify the pathway by which NO regulates K_ATP _channels in DRG neurons, using specific pharmacological tools at different levels of the cascades.

## Results

In recordings of native K_ATP _channels, we used 91 male rats: 48 controls (SS) that showed 0% probability of hyperalgesia response, and 43 rats with 46.1 ± 18% probability of hyperalgesia response (p < 0.001 *vs. *SS) in the ipsilateral paw after SNL (SNL). From these rats, we studied 218 control (SS) neurons with diameter 43.5 ± 4.1 μm, that did not differ from the diameter of 196 axotomized (SNL) neurons (44.7 ± 6.5 μm; p = 1.0 *vs. *SS).

### Single channel characteristics of K_ATP _channels in large DRG neurons dissociated from SS and SNL rats

**Figure 1:**
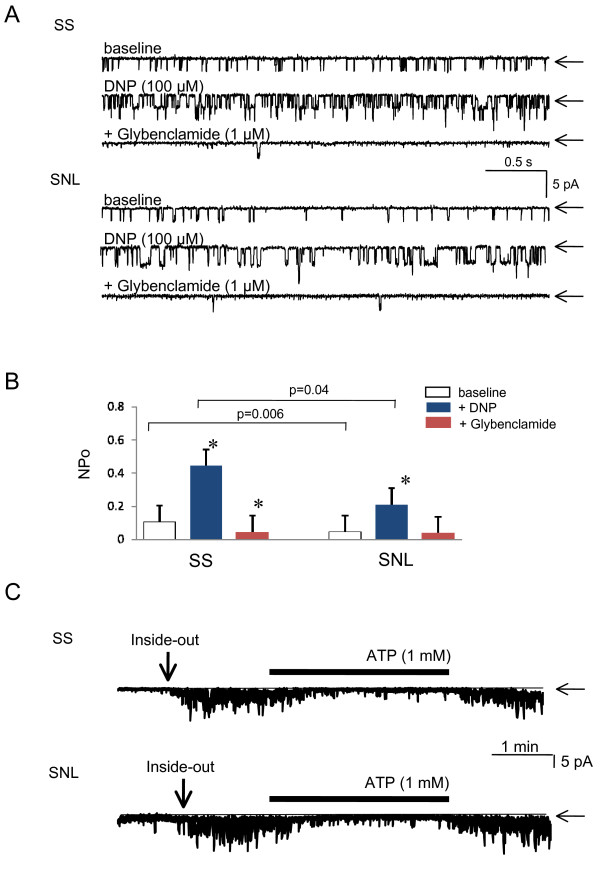
**Single-channel characteristics of K_ATP _channel in DRG neurons dissociated from SS and SNL rat**. (**A**) Representative current traces of K_ATP _channels in SS or SNL neurons recorded in cell-attached configuration at a holding potential of -60 mV. In SS and SNL neurons, bath application of DNP (100 μM) activated K_ATP _channel, and this channel activity was inhibited by glybenclamide (1 μM). The changes of NPo values are summarized in (**B**). Values are shown as mean ± SD (n = 8 each). (**C**) Representative K_ATP _current traces in SS or SNL neurons recorded in inside-out configuration at a holding potential of -60 mV. ATP (1 mM) was added to the intracellular solution as indicated by the horizontal solid bar. Arrows indicate closed channel state.

Single channel currents from either SS or SNL neurons were measured at -60 mV membrane holding potential, using cell-attached and inside-out patch clamp configurations. In cell-attached recordings, infrequent but significant spontaneous channel activity was observed in both control (SS) and SNL neurons (Figure 1A, baseline). However, this baseline activity was decreased in SNL neurons (NPo = 0.06 ± 0.02) compared with SS neurons (NPo = 0.12 ± 0.03; p = 0.006 *vs. *SNL; n = 8 in each group). Bath application of the uncoupler of mitochondrial ATP synthesis DNP (100 μM) gradually activated these baseline currents in both groups. However, DNP-induced channel opening was significantly reduced in SNL (NPo = 0.208 ± 0.16) compared to SS (NPo = 0.442 ± 0.26; p = 0.04 *vs. *SS; n = 8 in each group; Figure 1B). Addition of glybenclamide 1 μM, a specific K_ATP _channel inhibitor, almost completely blocked DNP-induced currents in both groups, indicating that these currents are conveyed via K_ATP _channels (p < 0.001 for each group). In inside-out patches, DNP (100 μM) had no direct effect on K_ATP _channels (p = 1.0 *vs*. baseline, n = 5 in each group).

In order to investigate the relative contribution of the intracellular milieu to regulation of channel properties, we next examined the K_ATP_channel behavior in excised inside-out membrane patches. When inside-out patch recordings at a holding potential of -60 mV were obtained in ATP-free solution, intense channel activity was observed in both groups. This channel activity was reversibly blocked by 1 mM of ATP (Figure 1C). The ATP sensitivity, tested by various intracellular ATP concentrations, was not significantly different between SS (IC_50 _= 14.7 μM; n = 5) and SNL (IC_50 _= 18.1 μM; n = 5; p = 1.0 *vs. *SS). In addition, single channel conductance in the presence of 100 μM ATP was not significantly different between SS (69.8 ± 12 pS; n = 5) and SNL (73.2 ± 7.1 pS; n = 5; p = 1.0 *vs. *SS). These results suggest that axotomy by SNL does not affect directly the properties of the K_ATP _channels.

### NO donor SNAP activates K_ATP _channels in large DRG neurons dissociated from control or SNL rats

**Figure 2:**
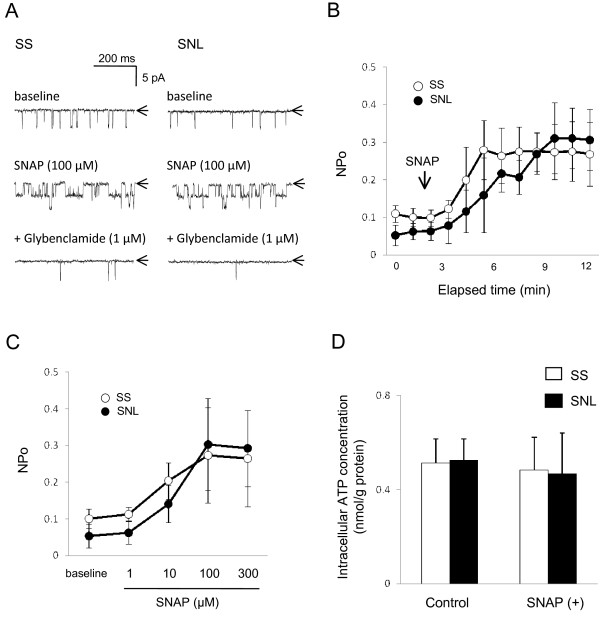
**Effects of SNAP on K_ATP _channel activity and intracellular ATP concentrations in DRG neurons**. (**A**) Representative current traces in SS or SNL neurons recorded in cell-attached configuration at a holding potential of -60 mV. In SS and SNL neurons, bath application of NO donor, SNAP (100 μM), activated K_ATP _channels. This channel activity was inhibited by glybenclamide (1 μM), a specific K_ATP _channel blocker. Arrows indicate closed channel state. (**B**) Time-dependent changes of SNAP-induced channel activity (NPo) in cell-attached recording, indicating the elapsed time to peak effect. Each point represents measurements from 8 patches (mean ± SD). (**C**) The concentration-dependent K_ATP _channel activation (NPo enhancement) induced by increasing SNAP concentrations in SS (open circle) and SNL (closed circle) neurons. Each point represents measurements from 8–9 patches (mean ± SD). (**D**) Intracellular ATP concentration measured by a luciferin-luciferase assay. Cells were stimulated for 10 min in the presence or absence of SNAP. Values are shown as mean ± SD (n = 5–6).

We tested the effects of NO donor SNAP on K_ATP _channel activity during cell-attached recordings from large DRG neurons dissociated from SS or SNL rats (Figure 2A). Bath application of SNAP (100 μM) significantly activated potassium currents in both SS and SNL neurons to a similar degree at steady state. Despite baseline difference, NPo steady-state values 10 minutes after application of SNAP were 0.27 ± 0.07 in SS and 0.31 ± 0.09 in SNL (p = 0.364; n = 7 in each group; Figure 2B). Oxidized SNAP (100 μM), which no longer releases NO [47], produced no significant activation of any potassium current either in SS (NPo = 102.3 ± 9.2% from baseline) or in SNL group (NPo = 97.0 ± 7.9% from baseline; n = 4 in each group). This discrepancy between regular and oxidized SNAP indicates that SNAP-induced current activation is dependent on release of NO from SNAP. SNAP-induced channel activity was blocked by subsequent addition of 1 μM glybenclamide in both neuronal groups, identifying the underlying conductance as K_ATP _current. The unitary channel amplitude was not different in any experimental condition in either neuronal group, indicative of unaltered channel conductance (Figure 2A). Effects of SNAP were reversible after washout periods of 2–5 min (data not shown). In addition to SNAP, another NO donor, SNP (10 μM), produced similar activation of K_ATP _channels in both SS and SNL neuronal populations. Specifically, NPo values 10 minutes after application of SNP were 0.24 ± 0.03 in SS (n = 3) and 0.26 ± 0.12 in SNL (n = 3) (p = 1.0).

SNAP gradually activated K_ATP _channels in both groups, in comparable time courses (Figure 2B). In the absence of SNAP, K_ATP _channel activity was stable over time in cell-attached configuration (data not shown). In both neuronal groups, SNAP activation of K_ATP _channels was concentration dependent, reaching a saturating plateau at a concentration of 100 μM or greater (Figure 2C).

Axotomy increases the excitability of DRG neurons, which consequently may change metabolic status of neurons. In addition, NO decreases intracellular ATP levels by inhibition of mitochondrial respiration in several cell types [48,49]. Therefore, we investigated whether axotomy or SNAP might alter the intracellular ATP concentrations in DRG neurons. Specifically, baseline intracellular ATP concentration did not differ between SS neurons (0.50 ± 0.10 nmol/g protein; n = 6) and SNL neurons (0.48 ± 0.13 nmol/g protein; n = 6; p = 1.00 *vs. *SS) (Figure 2D). SNAP also had no effect on intracellular ATP concentration 10 minutes after stimulation in DRG neurons of either SS rats (0.48 ± 0.14 nmol/g protein; n = 5; p = 1.00) or SNL rats (0.47 ± 0.19 nmol/g protein; n = 6; p = 1.00) (Figure 2D). These data suggest that SNAP-induced K_ATP _channel activation occurs independently of changes in intracellular ATP concentration induced by either axotomy or SNAP itself in DRG neurons.

### Endogenous NO does not affect baseline activity of K_ATP _channels

**Figure 3:**
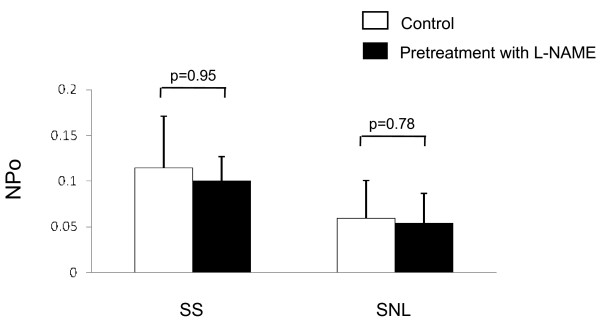
**Effects of endogenous NO on baseline K_ATP _channel activity in DRG neurons**. Baseline K_ATP _channel activity (NPo) was recorded 20 min after pretreatment with or without L-NAME (100 μM), a non-selective endogenous NO synthetase inhibitor. Each vertical barrepresents measurements from 6–7 cell-attached patches at a holding potential of -60 mV (mean ± SD). NS = no significant.

In normal adult mammals, nNOS is expressed in a few small- and medium-diameter rat lumbar DRG neurons [50]. However, nNOS expression is substantially increased after peripheral nerve axotomy [51]. Although the distribution and role of nNOS in large DRG neurons remain unclear, endogenous NO produced via nNOS may affect baseline activity of K_ATP _channel in this cell population. Therefore, we investigated the effect of endogenous NO on channel activity using L-NAME, an endogenous NOS inhibitor. Pretreatment with L-NAME (100 μM) for 20 minutes had no significant effect on baseline activity of K_ATP _channels compared to control (vehicle only, without L-NAME) in either SS (NPo after pretreatment with L-NAME = 0.114 ± 0.06 *vs. *0.105 ± 0.02 after vehicle; p = 0.95; n = 7 in each group) or SNL neurons (NPo after L-NAME = 0.06 ± 0.04 *vs*. 0.04 ± 0.03 after vehicle; p = 0.78; n = 7) (Figure 3). In inside-out patches, L-NAME had no direct effect on K_ATP _channels (n = 4, p = 0.975 *vs*. baseline). These results indicate that endogenous NO, possibly produced by nNOS in DRG neurons, does not affect basal K_ATP _channel activity in either SS or SNL neurons.

### 8-Br-cGMP activates K_ATP _channels, but fails to mimic completely the activation induced by NO

**Figure 4:**
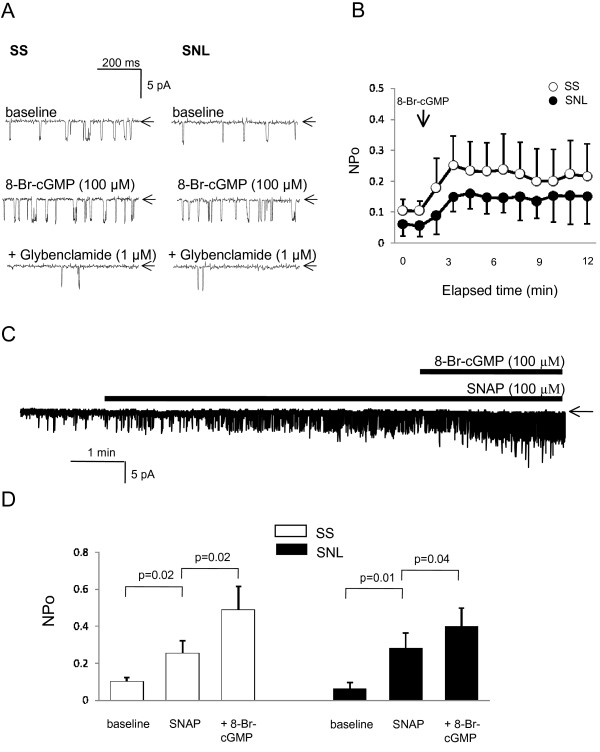
**Effects of 8-Br-cGMP on K_ATP _channel activity in DRG neurons**. (**A**) Representative current traces in SS and SNL neurons recorded from cell-attached configuration at a holding potential of -60 mV. Bath application of 8-Br-cGMP, a membrane-permeable analogue of cGMP, increased K_ATP _current in either SS or SNL neurons. Arrows indicate closed channel state. (**B**) Time dependent of 8-Br-cGMP-induced channel activation (NPo) during cell-attached recording, indicating the elapsed time to peak effect. Each point represents 8 SS and 7 SNL neurons. (**C**) Additional effect of 8-Br-cGMP on SNAP (100 μM)-induced steady-state K_ATP _currents in SS neurons. Changes of NPo values in either SS or SNL neurons are summarized in (**D**). Each vertical bar represents measurements from 5 patches (mean ± SD).

K_ATP _channels in cardiomyocytes, vascular or non-vascular smooth muscle cells, and pancreatic β cells are modulated by NO [52-56], indirectly via a classical cGMP-dependent pathway [52,53]. To investigate this pathway in large DRG neurons, we examined the effect of a membrane permeable cGMP analogue, 8-Br-cGMP, on neuronal K_ATP _channel activity in cell-attached patch recordings. Similar to SNAP, bath application of 8-Br-cGMP (100 μM) significantly activated K_ATP _channels in either SS neurons (NPo value 5 min after application of 8-Br-cGMP = 0.22 ± 0.08; n = 8; p = 0.002 *vs. *baseline) or SNL (NPo = 0.17 ± 0.06; n = 7; p = 0.001 *vs. *baseline) neurons. Subsequent addition of glybenclamide (1 μM) almost completely inhibited these currents (Figure 4A). Steady-state activation could be observed within only 2–3 minutes after exposure to 8-Br-cGMP (Figure 4B). When 8-Br-cGMP was applied after SNAP-induced steady-state activation had occurred (at least 5 min after SNAP), additional further channel opening was observed, both in SS neurons (n = 5; p = 0.02 *vs. *SNAP alone) and in SNL neurons (n = 5; p = 0.04 *vs. *SNAP alone) (Figure 4D). These data imply that NO does not activate K_ATP_channels via a cGMP-dependent PKG pathway, but via an alternative pathway. In order to examine the role of endogenous cGMP in activating K_ATP _channels, we also tested the effect of zaprinast, a specific PDE inhibitor, in cell-attached recordings. In these experiments, zaprinast had no effect on K_ATP _channel activity either in control neurons (n = 5; p = 0.88 *vs. *baseline), or in SNL neurons (n = 4; p = 1.0 *vs. *baseline) (data not shown).

### Effects of inhibitors of the NO/cGMP/PKG pathway on NO-induced K_ATP _channel activation

To further investigate whether NO activates K_ATP _currents via the classical NO/sGC/cGMP/PKG signaling pathway, we tested the effects of ODQ, a selective inhibitor of sGC, or the effects of KT 5823, a selective inhibitor of PKG, on SNAP-induced K_ATP _channel activity in large SS or SNL DRG neurons, using the cell-attached patch-clamp configuration.

**Figure 5:**
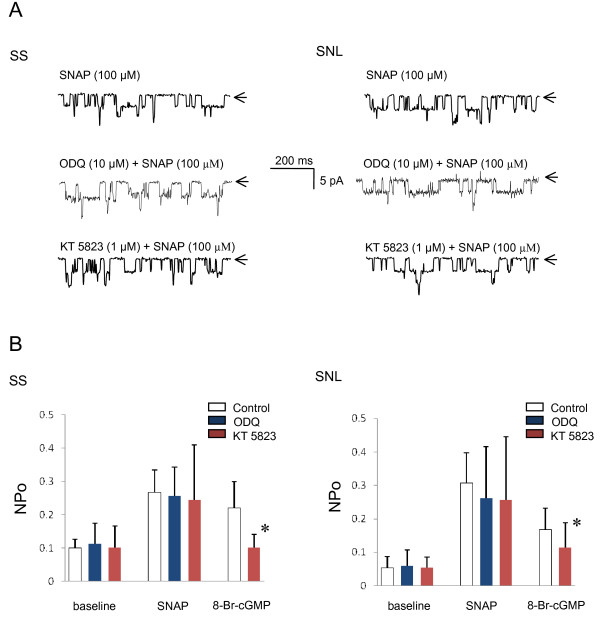
**Effects of NO/cGMP/PKG pathway inhibitors on NO-induced K_ATP _channel activity in DRG neurons**. (**A**) Representative traces of K_ATP _currents in cell-attached patches at -60 mV holding potential from SS or SNL neurons. SNAP (100 μM) activated channel opening (upper traces). Activating effect of SNAP was not inhibited by pretreatment for at least 20 min with neither ODQ (10 μM), a specific sGC inhibitor (middle traces), or KT5823 (1 μM), a PKG inhibitor (lower traces), in either SS or SNL neurons. Arrows indicate closed channel state. (**B**) Changes of NPo values obtained during baseline, after application of SNAP (100 μM), or 8-Br-cGMP (100 μM) with or without pretreatment of inhibitor in SS or SNL neurons. Each vertical bar represents measurements from 5–6 patches (mean ± SD).

Pretreatment with ODQ (10 μM) for 20 min failed to inhibit SNAP-induced K_ATP _channel activation in both SS neurons (NPo 10 min after SNAP = 0.25 ± 0.09; n = 6; p = 1.0 *vs. *control) and SNL neurons (NPo 10 min after SNAP = 0.27 ± 0.16; n = 6; p = 1.0 *vs. *control; Figure 5A, B). Similarly, pretreatment with KT 5823 (1 μM) for 20 min did not block SNAP-induced K_ATP _channel activation in either SS neurons (NPo 10 min after SNAP = 0.14 ± 0.17; n = 5; p = 1.0 *vs. *control) or SNL neurons (NPo 10 min after SNAP = 0.26 ± 0.19; n = 6; p = 1.0 *vs. *control; Figure 5A, B). These results suggest that NO activates K_ATP _channels via mechanism(s) other than the NO/cGMP/PKG pathway. Pretreatment with KT 5823 significantly reduces 8-Br-cGMP-induced K_ATP _channel activation in both SS neurons (n = 8; p < 0.001 *vs. *control) and SNL neurons (n = 6; p < 0.001 *vs. *control). These findings indicate that 8-Br-cGMP activates K_ATP _channels in both SS and SNL neurons via the activation of PKG. In inside-out patches, neither ODQ nor KT 5823 had any direct effect on K_ATP _channels (n = 4; p = 0.823 or n = 4; p = 0.295 *vs*. control, respectively).

### Direct effect of NO on K_ATP _channels in excised inside-out patches

**Figure 6:**
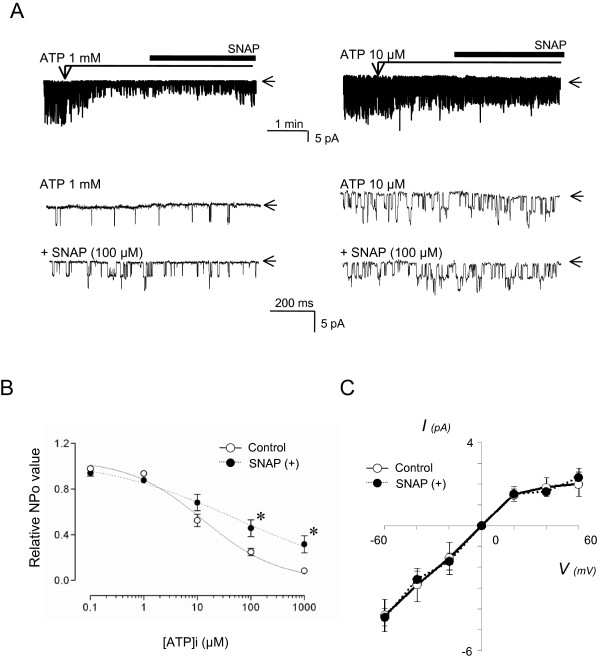
**Direct effects of NO on K_ATP _channel activity in SS DRG neurons**. (**A**) Representative current traces of K_ATP _channel activity in inside-out patches at -60 mV holding potential excised from SS neurons. Bath application of SNAP (100 μM) activated K_ATP _channel only in the presence of 1 mM, but not 10 μM [ATP]i. Arrows indicate closed channel state. (**B**) Concentration-response relationship between [ATP]i (0–1000 μM) and relative NPo in the presence or absence of SNAP in inside-out patches. The relative NPo values were calculated by dividing the channel activity (expressed as NPo) in the presence of various [ATP]i with the activity in the absence of ATP. Each point represents measurements from 5–6 patches (mean ± SD). *: indicate significant difference from control. (**C**) Current-voltage relations were plotted from inside-out recordings of single K_ATP _currents in the presence or absence of SNAP (100 μM) at membrane potentials between -60 and +60 mV (n = 5 each). Application of SNAP to patches did not affect single channel conductance.

To test the possibility of a direct effect of NO on K_ATP _channels in large DRG neurons, we next examined its effects of SNAP in excised inside-out patches under cell-free conditions (Figure 6A). When the patch was excised into a nucleotide-free solution, marked current activity was observed. This current was inhibited by [ATP]i 1 mM or 10 μM in a concentration-dependent fashion. In the presence of 1 mM [ATP]i, subsequent application of SNAP (100 μM) gradually activated K_ATP _channel (n = 6; p = 0.02 *vs. *baseline). However, in the presence of 10 μM [ATP]i, SNAP did not produce any significant channel activation (n = 6; p = 0.88 *vs. *baseline). These results indicate that direct K_ATP _channel activation by NO is [ATP]i-dependent.

We next tested the reciprocal interaction by testing whether NO-donor SNAP alters the ATP-sensitivity of K_ATP _channels. In the presence or absence of SNAP, various ATP concentrations (0 – 1000 μM) were applied in excised patches whereupon K_ATP _channel was suppressed in variable degrees (Figure 6B). [ATP]i, at concentrations = 100 μM, was less able to suppress K_ATP _channel activation in the presence of SNAP. Altered ATP sensitivity was evident by the five-fold rightward shift of the IC_50 _value for [ATP]i from 12.8 μM (n = 7) to 54.4 μM (n = 7). Baseline current amplitude and conductance of channels in SS neurons remained unchanged during patch exposure to 100 μM SNAP (n = 5 each, p = 0.59; Figure 6C). Similar direct effects of SNAP on ATP sensitivity and current amplitude/voltage relationship were also observed in SNL neurons (data not shown).

**Figure 7:**
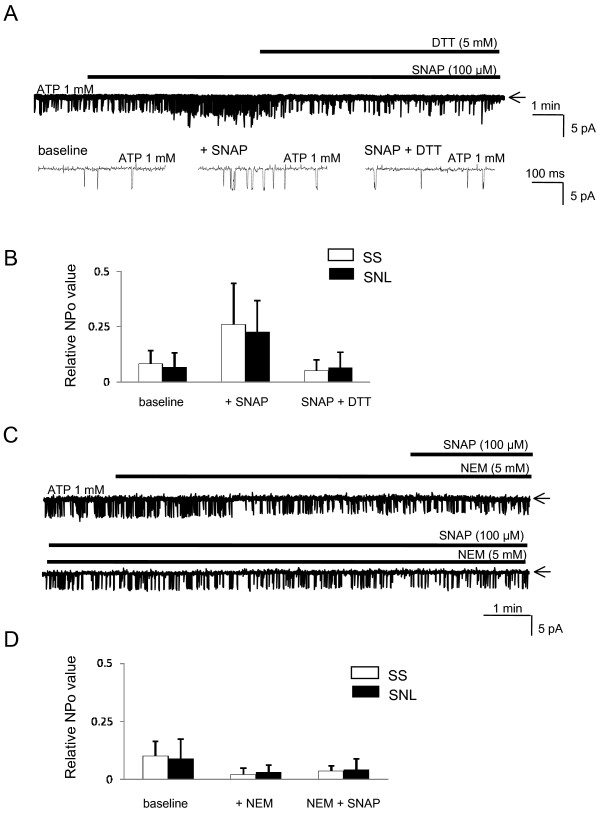
**Effects of thiol-modifying agents on NO-induced K_ATP _channel activation**. (**A**) Representative current traces in inside-out patches at -60 mV holding potential in the presence of 1 mM [ATP]i in large SS neurons. SNAP (100 μM) and DTT (5 mM), a thiol-reducing agent, were added to bath solution as indicated by the horizontal solid bars. Arrows indicate closed channel state. The changes of NPo values are summarized in (**B**). Values are shown as mean ± SD (n = 7 in each group). (**C**) Representative current traces in inside-out patches at -60 mV holding potential in the presence of 1 mM [ATP]i in large SS neurons. NEM (5 mM), a thiol alkylating agent, and SNAP (100 μM) were added to bath solution as indicated by the horizontal solid bars. Arrows indicate closed channel state. The changes of NPo values are summarized in (**D**). Values are shown as mean ± SD (n = 6 in each group).

A known alternative pathway for biological effects of NO is by direct S-nitrosylation of the critical cysteine thiol group(s) of target proteins [1]. To test whether this pathway is involved in direct modulation of K_ATP _channels by NO, we examined the effects of DTT, a thiol-specific reducing agent that reduces the nitrosylation by NO, on direct K_ATP _channel activation by SNAP. SNAP-induced K_ATP _channel activation in SS neurons in the presence of 1 mM [ATP]i was completely reversed by subsequent bath application of DTT (5 mM) (n = 7, p = 0.009; Figure 7A). In inside-out patches, DTT alone had no direct effect on K_ATP _channel (n = 5, p = 1.0 *vs. *baseline). Similar results were also observed in SNL neurons (n = 7, p = 0.01; Figure 7B).

To further test the possibility of S-nitrosylation by NO, we next tested the effects of NEM, which is known to covalently modify protein sulphhydryl groups making them incapable of nitrosylation, on SNAP-induced K_ATP _channel activation. Bath application of NEM (5 mM) to inside-out patches in the presence of 1 mM [ATP]i significantly decreased the basal channel activity (n = 6, p = 0.03 *vs. *baseline; Figure 7C). Presence of NEM completely eliminated the activating effect of SNAP on K_ATP _channels (n = 6, p = 1.0 *vs. *before SNAP; Figure 7D). Similar results were also observed in SNL neurons (n = 6, p = 1.0 *vs. *before SNAP; Figure 7D). These findings suggest that SNAP effects are most probably mediated via a redox switch, involving direct S-nitrosylation of K_ATP _channels. Furthermore, these direct actions of SNAP on K_ATP _channels were not altered by SNL, suggesting that the ability for S-nitrosylation to K_ATP _channel is preserved following nerve injury.

### Site of action of NO on recombinant K_ATP _channels expressed in COS7 cells

To determine the site of action of NO on K_ATP _channels, we investigated the effect of SNAP in inside-out recordings from cloned SUR1/Kir6.2 channels, the predominant K_ATP _channel in DRG neurons [57], heterologously expressed in COS7 cells. Transfected cells were identified by GFP co-transfection.

**Figure 8:**
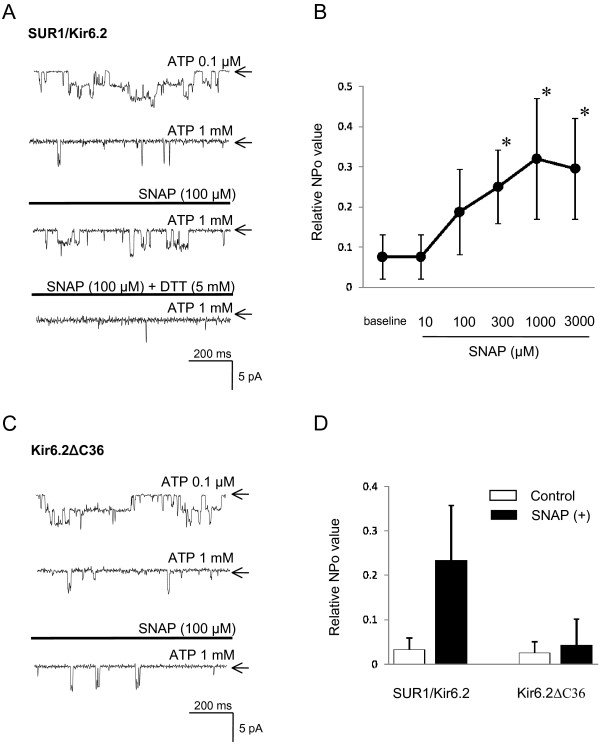
**Direct effect of NO on wild-type SUR1/Kir6.2 and truncated Kir6.2ΔC36 channels expressed in COS-7cells**. **(A**) Representative current traces of wild-type SUR1/Kir6.2 currents in inside-out patches at -60 mV holding potential. In the presence of 1 mM [ATP]i, bath application of SNAP (1 mM) activated wild-type SUR1/Kir6.2 channel. Activation was suppressed by the subsequent application of the thiol-reducing agent, DTT (5 mM). Arrows indicate closed channel state. (**B**) The concentration-dependent wild-type SUR1/Kir6.2 channel activity (NPo) on SNAP. Each point represents measurements from 7 patches (mean ± SD) *: indicate significant difference from baseline. (**C**) Representative traces of current via truncated Kir6.2ΔC36 channels in inside-out patches at -60 mV holding potential. In the presence of 1 mM [ATP]i, bath application of SNAP (1 mM) did not significantly activate truncated Kir6.2ΔC36 currents (in contrast to wild type SUR1/Kir6.2 currents shown in Figure 8A). Arrows indicate closed channel state. (**D**) Summary of SNAP effects on relative channel activity of wild-type SUR1/Kir6.2 currents and truncated Kir6.2ΔC36 channels, derived from experiments shown in Figure 8A and C. Each vertical bar represents measurements from 6–7 patches (mean ± SD).

We first investigated whether the effect of NO on cloned SUR1/Kir6.2 mimicked the NO effect on native K_ATP _channels in large DRG neurons. When the patch was excised into a low [ATP]i (0.1 μM) solution, recombinant SUR1/Kir6.2 channels showed marked current increases (Figure 8A; upper trace), that were strongly inhibited by 1 mM [ATP]i, confirming expression of functional K_ATP _channels. Subsequent addition of SNAP 1 mM into the bath significantly activated SUR1/Kir6.2 channels (n = 7, p < 0.001) in a concentration-dependent fashion (Figure 8B). These currents were almost completely suppressed by DTT (5 mM) (p < 0.001).

We next explored whether the site at which NO interacts with the K_ATP _channel lies on the regulatory (SUR1) or the pore-forming (Kir6.2) subunit, using a truncated isoform of Kir6.2 (Kir6.2ΔC36) that produces functional channels independently of SUR1 [58]. SNAP 1 mM fails to activate Kir6.2ΔC36 currents (n = 6, p = 1.0) in the presence of 1 mM [ATP]i (Figure 8C and 8D). This observation, in contrast to activation of wild type SUR1/Kir6.2 channels, indicates that NO interacts with the SUR1 subunit, rather than the Kir6.2 subunit.

### Effects of cysteine mutation within nucleotide-binding domain of SUR1

The [ATP]i-dependent modulation of native K_ATP _channel by NO (Figure 6B) suggests that cysteine residues reacting with NO might be located at ATP-binding sites of SUR1 subunit; specifically within NBD1 and/or NBD2. In addition, the highly conserved Walker A (ATP-binding) motifs of SUR1 contain one single cysteine residue, at position 717 (C717) in NBD1. The ability of a thiol-modifying (alkylating) agent, NEM, (which inhibits native K_ATP _channels), to prevent ATP binding at NBD1 is abolished by mutation of C717 [59]. This implies that cysteine at position C717 may be redox-active. We therefore focused on this cysteine residue, and examined the effect of mutating C717 to serine (C717S) on the sensitivity of the cloned SUR1/Kir6.2 channel to NO.

**Figure 9:**
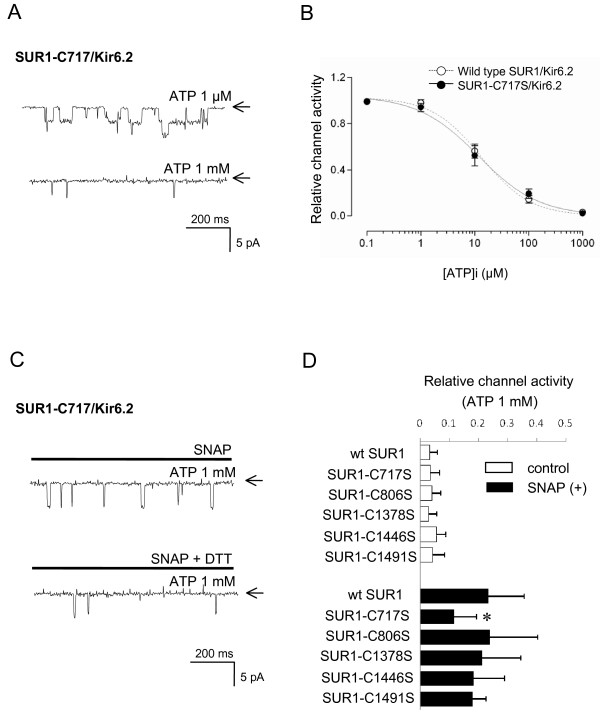
**Effects of mutations within nucleotide-binding domain of SUR1 on channel activation by NO**. (**A**) Representative traces of currents via SUR1-C717S/Kir6.2 channels in inside-out patches at -60 mV holding potential. Bath application of ATP (1 mM) inhibited SUR1-C717S/Kir6.2 channels. Arrows indicate closed channel state. (**B**) Concentration-response relationship curves between [ATP]i and relative NPo values for wild-type SUR1/Kir6.2 or SUR1-C717S/Kir6.2 channels. The relative channel activities were calculated by dividing the channel activity in the presence of various [ATP]i with the activity in the absence of ATP. Each point represents measurements from 7 patches (mean ± SD). (**C**) Representative current traces of SUR1-C717S/Kir6.2 channel currents in inside-out patches at -60 mV holding potential. In the presence of 1 mM [ATP]i, bath application of SNAP (1 mM) activated SUR1-C717S/Kir6.2 channel, but less than wild-type SUR1/Kir6.2 channels. Activation of these currents was suppressed by the subsequent application of a thiol-reducing agent, DTT (5 mM). Arrows indicate closed channel state. (**D**) Summary of ATP-sensitivity and SNAP effects on relative NPo values of wild-type (control) and mutated SUR1/Kir6.2 currents. The relative channel activities were calculated by dividing the channel activity in the presence or absence (1 mM [ATP]i only) of SNAP (100 μM) with the activity in the absence of ATP. Each horizontal bar represents measurements from 7–8 patches (mean ± SD).

A patch containing recombinant SUR1-C717S/Kir6.2 channels, also showed marked current increases when excised into a low [ATP]i (0.1 μM) solution (Figure 9A). These currents were strongly inhibited by 1 mM [ATP]i (n = 7, p < 0.001). The ATP-sensitivity of SUR1-C717S/Kir6.2 channels (IC_50 _= 13.1 μM, n = 7) was not significantly different from that of wild type SUR1/Kir6.2 channels (IC_50 _= 11.6 μM, n = 7). Similar to the response of wild type SUR1/Kir6.2 channels, bath application of 1 mM SNAP significantly activated the SUR1-C717S/Kir6.2 channel in the presence of 1 mM [ATP]i. However SNAP was significantly less potent in activating the SUR1-C717S/Kir6.2 channel compared with the wild-type SUR1/Kir6.2 channel (n = 8, p < 0.02 vs. wild type; Figure 9C). SNAP-induced SUR1-C717S/Kir6.2 channel activation was reversed by DTT (5 mM) (n = 7).

In addition to C717S, we further examined the activating effects of NO after mutating the cysteine residues within NBD1 and NBD2 to serine, including those cysteine residues adjacent to C717 within NBD1 (C806S), and all three cysteine residues within NBD2 (C1378, C1446, C1491). All these mutated channels showed similar ATP-sensitivity (Figure 9D, controls). Among these mutants, only SUR1-C717S/Kir6.2 channels significantly affected NO-sensitivity compared with wild type SUR1/Kir6.2 channels.

## Discussion

**Figure 10:**
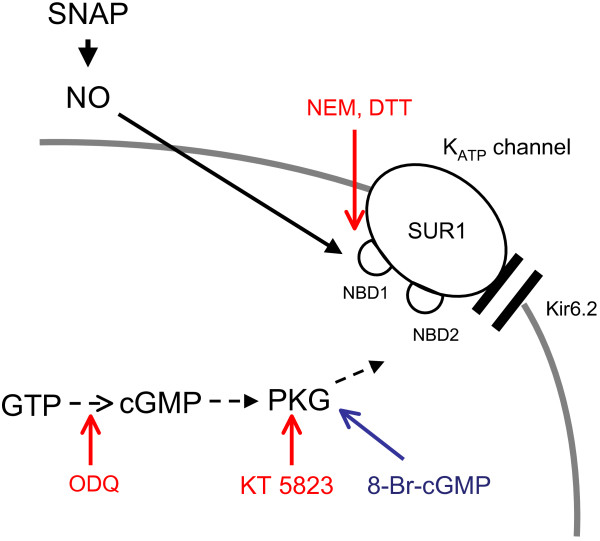
**Possible pathways involved in K_ATP _channel activation by NO and sites of action of modulators**. SNAP exogenously releases NO that might have activated sGC by diffusion into the cytosol. sGC in other tissues generates endogenous cGMP, which may further activate PKG in order to produce downstream effects on the channel. However, the results of this study imply that sGC is not active in DRG neurons. Dashed arrows indicate also that the indirect NO/sGC/cGMP/PKG pathway is not active in DRG neurons, because ODQ, a specific sGC inhibitor, and KT 5823, a specific PKG inhibitor, fail to block it (red arrows). In contrast, exogenous membrane permeable 8-Br-cGMP (blue arrow) activates K_ATP _channels via PKG. Solid arrow indicates that NO most likely activates K_ATP _channels via S-nitrosylation on the NBD1 within the SUR1 subunit. This direct pathway is prevented by thiol-alkylating, or reversed or thiol-reducing agents (NEM or DTT, respectively; red arrow).

Our findings demonstrate that NO activates K_ATP _channels in mammalian sensory neurons. We additionally showed that inhibition of sGC and PKG fails to block the activation by NO, indicating that NO signaling is not through the classical indirect pathway (Figure 10). NO-induced activation of K_ATP _channels also occurs in cell-free patches, showing that cytosolic elements are not needed for NO action. However, NO activation of K_ATP _current is inhibited by thiol-reducing or thiol-alkylating agents, which demonstrates that S-nitrosylation is needed for NO action (Figure 10). Since NO fails to activate current conveyed by Kir6.2 channels that lack SUR1, the NO effect must be sited on the SUR1 subunit. Mutagenesis of cysteine residues within SUR1 limits NO-induced K_ATP _channel activation, confirming SUR1 as the site of S-nitrosylation. Taken together, these novel findings prove that NO activates K_ATP _channels via S-nitrosylation of one or more cysteine residues on the SUR1 subunit (Figure 10).

Although the sGC/cGMP/PKG pathway mediates NO regulation of K_ATP _channels in cardiomyocytes, vascular and non-vascular smooth muscle cells, and pancreatic β cells [52-56], this is not the case in DRG neurons. Our finding that NO/sGC/cGMP is inactive in DRG cells is consistent with reports indicating the absence of sGC in mice [45] and cGMP in rat DRG neurons [46] using immunohistochemical techniques. However, our data show that an exogenous membrane permeable cGMP analogue still activates K_ATP _channels via a PKG-dependent mechanism, as in other tissues [53,60]. The presence of PKG in DRG neurons that lack an up-stream NO/sGC/cGMP pathway suggests an alternative pathway of activation, perhaps via natriuretic peptide receptor-B stimulation [45]. PKG expression is not altered by axotomy [61], possibly explaining our findings of similar PKG-dependent 8-Br-cGMP actions in both control and axotomized neurons.

As an alternative to the indirect cGMP-signaling pathway, NO can also directly modify proteins by S-nitrosylation, through which S-nitrosothiols (SNOs) are formed by covalent addition of an NO moiety (formally as NO^+^) to cysteine residues. S-nitrosylation signaling underlies NO modification of many ion channels, including the NMDA receptor-channel complex, Ca^2+^-activated K^+ ^channels, Na^+ ^channels in baroreceptors, cardiac Ca^2+ ^release channels, and olfactory cyclic nucleotide-gated channels [1,62-65]. Changes in the thiol-redox status of cysteine residues by thiol-group-modifying substances are also involved in NO modulation of K_ATP _channels in pancreatic β cells, cardiomyocytes, and skeletal muscle cells [66-68]. Our new observation adds sensory neurons as a site at which S-nitrosylation of cysteines on the K_ATP _channel activates the K_ATP _currents.

In contrast to our findings, NO has no direct effect on native cardiac and pancreatic K_ATP _channels [53,55]. Although the mechanisms determining susceptibility to S-nitrosylation by NO remain unknown, access of NO to available cysteine targets is determined by peptide tertiary structures and associated proteins [1]. Also, the number and location of cysteines may vary between K_ATP _channels of different subunit compositions [20].

As in native K_ATP _channels, we showed that NO directly activates cloned SUR1/Kir6.2 channels expressed in COS7 cells, although this effect is approximately 3-fold less potent than in native channels. Compared to SUR1/Kir6.2 expressed in DRG neurons [57], the decreased sensitivity of channels in COS7 cells to NO may reflect differences in technique (inside-out recordings from cloned channels, cell-attached from native K_ATP _channels), or variations in the redox status of the cytosolic environment, and different protein conformation in the two experimental models. Despite these differences, we observed direct modulation of K_ATP _channels by NO in both settings.

Previous studies using mutagenesis have revealed that thiol-reagents modify K_ATP _channel activity by interacting with a single cysteine residue at either Kir6.2 or SUR1. Oxidizing agents pCMPS and MTSET interact with a specific single cysteine residue on Kir6.2, whereas the alkylating agent NEM interacts with a cysteine residue on SUR1 [59,69,70]. In the present study, NO failed to modulate Kir6.2 channels lacking SUR1, indicating that NO does not act on a thiol oxidizing-sensitive site on Kir6.2, but that the critical cysteine residue is on the SUR1.

Nucleotides regulate K_ATP _channel activity in a complex fashion. The channel is inhibited by ATP binding to Kir6.2, whereas the channel is activated by Mg-nucleotides (MgATP, MgADP) interacting with the two NBDs on SUR1 [20-22]. Thus [ATP]i-dependence of activation of native K_ATP _channels by NO suggests that the target cysteine is located at the NBDs within SUR1. Furthermore, our site-directed mutagenesis demonstrated that only mutation of cysteine residue at position 717 (C717) within NBD1 significantly inhibited the activation by NO. Cys717 is the sole cysteine residue located on the highly conserved Walker A (ATP-binding) motifs of SURI. Because the alkylating agent NEM directly interacts with this residue [59], it is likely that cysteine nitrosylation by NO modulates nucleotides-SUR1 interaction, resulting in allosteric activation of the K_ATP _channel. Mutation of Cys717 did not completely inhibit the NO-induced channel activation, suggesting that other cysteine residues besides those in the NBD may be involved in NO action via poly-nitrosylation, as has been shown in cardiac calcium release channel [65].

The significance of the K_ATP _channel activation by NO in DRG neurons is presently unknown. If DRG neurons lack sGC and the capacity to mobilize the NO/sGC/cGMP pathway, it is likely that S-nitrosylation is the only mechanism by which NO activates K_ATP _channels in these cells. nNOS expression in DRG neurons is markedly up-regulated after injury, especially in the small and medium sized neuronal population, enabling these neurons to produce endogenous NO [50,51]. Additionally, glial satellite cells also produce and release NO. Since this molecule is highly diffusible, NO from this sources may influence K_ATP _channels predominantly expressed in large DRG neurons. The direct S-nitrosylation pathway requires higher NO levels and responds more slowly than the indirect pathway, and thereby producing a threshold effect by which K_ATP _channels would respond only to intense NO stimuli and only in a prolonged temporal domain [1].

Our present data confirm our previous findings of K_ATP _current loss following nerve injury [25,35]. Since axotomy does not alter the channel biophysical properties or sensitivity to modulators, loss of K_ATP _current may be the result of regulatory shifts that reduce K_ATP _channel opening. An implication of our findings is that decreased channel opening after axotomy may be driven by diminished NO levels, but this needs to be confirmed by further studies. Our results also suggest that K_ATP _channel modulation by S-nitrosylation signaling is not altered by injury. Our present data show that activation by NO restores the K_ATP _current in axotomized neurons. Since K_ATP _currents decrease neuronal excitability and diminish excitatory neurotransmission, it is likely that elevation of channel opening by NO may produce analgesic effects even in conditions in which there is no preceding deficit of K_ATP _currents. We did not directly assess any attenuation in neuronal excitability mediated by increased activity of K_ATP _channels following application of NO, and this may be a limitation of this study. However, NO-regulation explains why some drugs exert peripheral analgesic effects via NO-related pathways that activate peripheral neuronal K_ATP _channels [15,17-19,39]. Our findings, along with previous studies, demonstrate that NO mediated activation of K_ATP _channels might be employed as a pathway for therapy against pain.

## Conclusion

NO exogenously released by the NO donor SNAP activates K_ATP _channels in DRG neurons. This activation is not altered by painful-like nerve injury. Effects mediated by NO do not involve the indirect sGC/cGMP/PKG signaling pathway, but may be the consequence of direct S-nitrosylation of cysteine residue(s) on SUR1 subunits associated with ATP-binding site.

## Methods

### Approval of experimental procedures

All procedures in this study were approved by the Institutional Animal Care and Use Committee (IACUC) of the Medical College of Wisconsin, Milwaukee, Wisconsin.

### Animal Surgery

For all experiments, we used Male Sprague-Dawley rats (125–160 g), 6 weeks old and weighing 125–160 g, which were obtained from a single vendor (Taconic Inc., Germanville, NY). We randomly assigned rats to a surgical axotomy group using the spinal nerve ligation (SNL) model [71], or to a control group that received sham skin operation (SS). We anesthetized rats with isoflurane (1.5–3% in oxygen) by spontaneous ventilation. We exposed the right lumbar paravertebral area through a lumbar incision, accessed the fifth (L5) and sixth (L6) lumbar nerves, which we ligated with 6-0 silk ligature, and transected distal to the ligature. We then closed the lumbar fascia with 4-0 absorbable vicryl polyglactin suture, and the skin with three to five surgical staples. Unlike the originally described method [71], we did not excise the paraspinous muscles or the adjacent articular processes. In control rats, we performed sham operation by lumbar skin incision and closure by staples only.

### Behavioral testing

We selected rats that successfully developed neuropathic pain behavior using a previously validated [72] and reported method [73] that identifies hyperalgesia after SNL with high specificity. We tested animals on the 10th, 12th, and 14th postoperative days onto a 0.25 in. wire grid. Briefly, after 30 min's rest, we touched the hind paws randomly using a 22-gauge spinal needle with pressure adequate to indent but not penetrate the skin (to a total of ten applications per session). Rats subjected to SS operation exhibited normal, brief reflexive withdrawal responses and were used as controls. Rats that displayed a probability of hyperalgesia-type response (> 2 s sustained lifting, licking, chewing, or shaking of the paw) at least 20% averaged over 3 test days, and normal contralateral responses after SNL, were considered as responders with neuropathic behavior after SNL. Behavioral testing was repeated on the day of study to confirm the presence of hyperalgesia. We further included only these responding rats in our study, in comparison with controls.

### Cell isolation and plating

We harvested control (L4 and L5) or axotomized (L5) ganglia, from control (SS) or hyperalgesic rats (SNL) respectively, between the 17^th ^and 28^th ^postoperative days. We sacrificed animals by decapitation under deep isoflurane anesthesia, excised ganglia through a lumbar incision, and at the same time confirmed accuracy of initial surgery.

For patch-clamp recordings, we placed excised DRG into separate 35 mm Petri dishes containing cold, oxygenated, Hanks Balanced Salt Solution (Gibko, Invitrogen, Grand Islan, NY), and minced them with iris scissors. We dissociated ganglia enzymatically in a solution containing 0.25 ml 0.05% w:v liberase blendzyme 2 (Roche Diagnostics Corp., Indianapolis, IN) and 0.25 ml DMEM/F12 with glutaMAX (Dulbecco's modified Eagle's medium F12; Gibco, Invitrogen Corp., Carlsbad, CA) in an incubator at 37°C for 30 min. We then removed the supernatant after centrifugation and re-incubated cells at 37°C for another 30 min in 0.2 ml 0.0625% trypsin (Sigma, St. Louis, MO) and 0.0125% deoxyribonuclease 1 (150,000 U, Sigma, St. Louis, MO) in 0.25 ml DMEM. We then isolated cells by centrifugation (600 rpm for 5 min) after adding 0.25 ml trypsin inhibitor 0.1% w:v (Sigma St. Louis, MO), and re-suspended them in a culture medium consisting of 0.5 mM glutamine, 0.02 mg/ml gentamicin (Gibco, Invitrogen Corp., Carlsbad, CA), 100 ng/ml nerve growth factor 7S (Alomone Labs, Jerusalem, Israel), 2% (vol/vol) B-27 supplement (Gibco, Invitrogen Corp., Carlsbad, CA), and 98% (vol/vol) neural basal medium A (Gibco, Invitrogen Corp., Carlsbad, CA). Finally, we plated cells onto poly-l-lysine-coated 12-mm glass coverslips (Deutsche Spiegelglas; Carolina Biologic Supply, Burlington, NC), kept them in a humidified incubator at 37°C with 95% air and 5% CO_2_, and studied them within 3–8 h of dissociation.

### Molecular Biology and Transfection

K_ATP _channel-deficient COS7 cells were plated at a density of 3 × 10^5^/dish (35 mm in diameter) and cultured in Dulbecco's modified Eagle's medium supplemented with 10% fetal calf serum. Rat Kir6.2 (GenBank X97041) and rat SUR1 (GenBank X97279) cDNAs were used for expression study. A truncated form of Kir6.2 lacking the last 36 amino acids at the C-terminus was obtained by polymerase chain reaction amplification. Polymerase chain reaction products were cloned into the pCR3.1 vector by using the TA cloning system (Invitrogen Corp., Carlsbad, CA, USA) and then cloned into the pcDNA3.1 (-) vector (Invitrogen Corp.) for mammalian expression. Point mutation of cysteine at position 717 (C717), C806, C1378, C1446, and C1491 within SUR1 to serine (C717S, C806S, C1378S, C1446S, and C1491S) was performed by using the Site-Directed Mutagenesis system (Invitrogen Corp.). All cDNA products were sequenced by using the BigDye terminator cycle sequencing kit and an ABI PRISM 377 DNA sequencer (Applied Biosystems, Foster City, CA) to confirm the sequence. A full-length Kir cDNA and a full-length SUR cDNA were subcloned into the mammalian expression vector pcDNA3.1 (-). For electrophysiological recordings, either wild-type or mutated pcDNA3.1 (-) Kir6.2 alone (1 μg), or pcDNA3.1 (-) Kir6.2 (1 μg) plus pcDNA3.1 (-) SUR1 (3 μg) were transfected into COS7 cells with green fluorescent protein cDNA as a reporter gene by using lipofectamine and Opti-MEN 1 reagents (Life Technologies Inc., Rockville, MD) according to the manufacturer's instructions. After transfection, cells were cultured at 37°C with 95% air and 5% CO_2 _for 48–72 h before being subjected to electrophysiological recordings.

### Measurement of intracellular ATP concentration

For measuring intracellular ATP concentration, we extracted intracellular ATP ([ATP]i) using ATP extraction kit (Toyo Ink, Tokyo, Japan). After preincubation in HBS for 30 min, we incubated DRG neurons in the presence or absence of 100 μM SNAP for 10 minutes. We quantitatively measured the [ATP]i level using the luciferin-luciferase assay solution (Toyo Ink), according to protocols provided by the manufacturer.

### Electrophysiological recordings

We visualized plated neurons using an inverted Nikon Diaphot 300 microscope with Hoffman modulation optic system. We selected only large diameter neuronal somata (≥ 40 μm in diameter) because we have previously shown that only these develop K_ATP _current alterations [35], as well as changes indicative of increased excitability after painful-like nerve injury by SNL [74]. Large neuronal somata, roughly correspond to large, myelinated Aβ fibers.

We recorded current passing through single channels in inside-out or cell-attached patch configurations. In cell-attached patches, both bath and pipette (extracellular) solutions were composed of the following: 140 mM KCl, 10 mM HEPES, 5.5 mM dextrose, and 1 mM EGTA. In inside-out patches, the bath (intracellular) solution contained 140 mM KCl, 1.2 mM MgCl_2_, 10 mM HEPES, 1.5 mM EGTA and 5.5 mM dextrose. The pipette (extracellular) solution was of the same composition as that used in cell-attached recordings. The pH of all solutions was adjusted to 7.4 with KOH. Osmolality was adjusted approximately to 300 mOsm/l by adding sucrose if necessary. We pulled patch micropipettes from borosilicate glass capillaries using a Flaming/Brown micropipette puller, model P-97 (Sutter, San Rafael, CA) and flame polished them with a microforge polisher (Narishige, Tokyo, Japan) prior to use. Their resistance ranged between 3 and 6 MΩ when filled with the internal solution, and placed into the recording solutions.

We recorded single channel currents at room temperature (20–25°C) using an Axon CNS Multiclamp 700B amplifier, digitized them using an analog-to-digital converter (Axon CNS DigiData 1440A; Axon Instruments, Foster, CA), and stored them into a PC. Sampling frequency of single-channel data was 5 KHz with a low-pass filter (1 KHz). We used pClamp version 10.2 software (Axon Instruments) for data acquisition and analysis. We applied a conventional 50% current amplitude threshold level criterion to determine open events. Channel open probability (Po) was determined from the ratios of the area under the peaks in the amplitude histograms fitted by a Gaussian distribution. We calculated channel activity as NPo (where N is the number of observed channels in the patch) from data samples of either 30 s (in inside-out recordings), or 60 s (in cell-attached recordings), in the steady state. In inside-out recordings, NPo of the K_ATP _channels was normalized to the baseline NPo value obtained before test drugs at bathing solution without ATP (indicating relative channel activity).

### Drugs

SNAP, SNP, glybenclamide, L-NAME, zaprinast, ODQ, KT5823, 8-Br-cGMP, DTT, and NEM were purchased from Sigma, St. Louis, MO, USA. SNAP was stored at -20°C as a 30 mM stock solution in methanol. The solution of SNAP was prepared 15 min before being tested experimentally, and was not used thereafter for longer than 2 hours. It was also continuously kept protected from light. Glybenclamide (10 mM) and ODQ (20 mM) were stocked in DMSO. SNP, L-NAME, DTT, and NEM were prepared as a 1 M, 10 mM, 0.5 M, and 50 mM stock solution in distilled water, respectively. All drugs were diluted in perfusate as indicated. Oxidized SNAP, that no longer generates NO, was prepared as described previously, by allowing SNAP dissolved in DMSO, to decompose at room temperature for 48 hours [47]. When added to the bathing solution, the maximal concentrations of either methanol (0.01%) or DMSO (0.01%) alone did not exert any affect, nor modify the KATP channel activity of the preparation. Glybenclamide [75], L-NAME [76], zaprinast [60], ODQ [77], KT5823 [60], DTT [62], and NEM [78] were applied at concentrations that have been previously reported as effective in inhibiting their respective targets.

### Data Analysis and Statistics

Data are expressed as means ± SD. The effect of bath changes, indicative of various drug effects, was evaluated using repeated measures ANOVA to identify main effects, followed by Bonferoni *post hoc *tests whenever appropriate. Student's t-tests were also used for pair-wise comparisons, whenever needed. Significance was accepted at *P *< 0.05. Concentration-response curves were plotted by non-linear regression using sigmoidal concentration-response (variable slope) equations (Y = bottom/(1+10^(logEC50-X)*Hill slope), and statistically compared using the Prism software. The SPSS statistical software was used for statistical analysis.

## Abbreviations

K_ATP_: ATP-sensitive; DRG: dorsal root ganglion; NO: nitric oxide; SNL: spinal nerve ligation; SS: sham surgery; [ATP]I: intracellular ATP concentration; sGC: soluble guanylate cyclase; SUR: sulfonylurea receptor; Kir6.2: inwardly-rectifying potassium channel 6.2; NBD: nucleotide binding domain; nNOS: neuronal nitric oxide synthase; DNP: 2,4-Dinitrophenol; SNAP: S-nitroso-N-acetylpenicillamine; SNP: sodium nitroprusside; L-NAME: NG-nitro-L-arginine methyl ester; ODQ: 1H-[1, 2, 4] oxadiazolo [4, 3-a] quinoxalin-1-one; 8-Br-cGMP: 8-bromo-cGMP sodium salt; DTT: di-thiothreitol; NEM: N-ethylmaleimide; DMSO: dimethyl sulfoxide.

## Competing interests

The authors declare that they have no competing interests.

## Authors' contributions

TK, MK, and CDS carried out the electrophysiological experiments, data analysis and wrote the manuscript as well as interpreted data. VZ, MYL, HEW, and GG participated in the electrophysiological experiments in DRG neurons. TK, VZ, HEW, GG, and JBM carried out animal surgery and behavior testing. TK and MK carried out molecular biology and ATP content analysis. WMK, QHH, and CDS conceived the study, and participated in its design and coordination. All authors have read and approved the final manuscript.
